# The Chemical Profiles and Antioxidant Properties of Live Fruit or Vegetable Vinegars Available on the Polish Food Market

**DOI:** 10.3390/foods13101488

**Published:** 2024-05-11

**Authors:** Klaudia Melkis, Karolina Jakubczyk

**Affiliations:** Department of Human Nutrition and Metabolomics, Pomeranian Medical University in Szczecin, 24 Broniewskiego Street, 71-460 Szczecin, Poland; 58147@student.pum.edu.pl

**Keywords:** vinegar, antioxidants, fermented product, polyphenols, fruit vinegar, vegetable vinegar, antioxidant properties

## Abstract

Live vinegar is a product formed through a two-step fermentation process of a sugar substrate that has not been subjected to filtration or pasteurization. This is considered to preserve all nutrients and biologically active microorganisms, making it a product with a valuable composition and beneficial properties. Therefore, the purpose of this study was to analyze the chemical composition and antioxidant properties of the selected vinegars available on the Polish food market. The material in the study consisted of four live (naturally turbid, unfiltered, unpasteurized) fruit or vegetable vinegars: apple, pear, rhubarb, and lemon. Spectrophotometric, HPLC, and GC methods were used. Among the vinegars tested, lemon vinegar had the highest vitamin C content—15.95 mg/100 mL. Apple vinegar proved to be the best source of polyphenols and flavonoids (TPC—191.97 mg GAE/L, TFC—70.22 mg RE/L). All of the vinegars contained dihydroxybenzoic acid, 4-hydroxybenzoic acid, caffeic acid, 2-hydroxycinnamic acid, and myricetin. The acetic acid content of the tested vinegars ranged from 29.180 to 38.125 mM/L. The pH values ranged from 3.14 to 3.41. In conclusion, the most promising nutraceutical with potentially beneficial health-promoting properties seems to be apple vinegar.

## 1. Introduction

Vinegar is a product formed through a two-step fermentation process of a sugar substrate, which can be almost any raw material that is a source of carbohydrates [1]. Firstly, as a result of the metabolic activity of yeast (usually *Saccharomyces cerevisiae*) under anaerobic conditions, the conversion of fermentable sugars to ethanol occurs [2]. Then, bacteria of the genus *Acetobacter* convert ethanol to acetic acid through oxidation [3,4].

To produce vinegars on an industrial scale, methods based on rapid fermentation processes are mainly used: drip (generator) and pit (in fermenters) [5,6]. In order to obtain the expected result as quickly as possible, the liquid is set in motion, enriched with cultures of specialized bacteria and additionally oxygenated, thanks to which the obtained vinegar is bottled several hours after the start of the process. In the traditional method, called surface fermentation, the raw material is subjected to fermentation, which takes place slowly, lasting up to several months. The longer fermentation period allows for the accumulation and formation of a cloudy gelatinous structure consisting of cellulose, yeast, and live bacterial colonies, called the mother of vinegar [7]. Although the vinegar formed in this way does not require pasteurization, as the acetic acid in it is a natural preservative, most producers subject it to filtration and pasteurization to obtain a product with greater clarity and a longer shelf life [8]. Nevertheless, unfiltered, and unpasteurized vinegar, often referred to as live vinegar, retains all its nutrients and biologically active microorganisms, making it a product with a valuable composition and potentially health-promoting properties [9]. Moreover, even after bottling, beneficial biochemical processes can take place in it, thanks to the presence of the mother of vinegar.

The virtues of vinegar were already appreciated in ancient civilizations, where it was not only a preservative or condiment, but also a medicine [10]. It was used to stimulate circulation, cleanse the blood, liver, and lymph nodes, improve vitality and support immunity. Egyptians also used it to treat mushroom poisoning and a lack of appetite. The father of medicine himself, Hippocrates, used vinegar in his practice as a disinfectant for wounds, while, for colds and coughs, he recommended a mixture of apple vinegar with honey and water, called oxymel [11]. Furthermore, vinegar was thought to benefit the skin and hair, hence, it was also used in cosmetics. Current knowledge indicates that the range of diseases and disorders whose development can be prevented or counteracted by consuming vinegar is much more extensive. These include cardiovascular disease, metabolic-associated fatty liver disease (MAFLD), and diabetes, among others [12,13,14,15]. Recent studies report that apple vinegar also shows promise in the treatment of osteoporosis in menopausal women, polycystic ovary syndrome (PCOS), insulin resistance, neurological disorders such as Alzheimer’s disease, and weight loss [16,17,18,19,20,21]. Vinegar’s invaluable role in psychological well-being has also been suggested, as demonstrated in a randomized clinical trial [22].

The multidirectional effects of vinegars are the result of the synergy of a wide range of functional substances such as organic acids, mainly acetic acid, fructooligosaccharides, amino acids, minerals, vitamins, color compounds, and fermentation products that give it its characteristic taste and aroma—esters, ketones, and aldehydes [23,24,25]. Vinegars are also characterized by the presence of several phytonutrients with antioxidant activity (e.g., gallic acid, caffeic acid, protocatechuic acid), which may be derived from the starting raw material or formed during the fermentation process [26,27].

However, the presence and quantity of nutrients in vinegars is not constant. A number of factors, such as the production technique employed, the fermentation parameters (including time, temperature, oxygen availability, and pH), the microbial composition involved in the fermentation process, the contact with the wall of the fermentation tank, the storage conditions and duration, and particularly the variability dependent on the raw materials used, influence the physicochemical and phytochemical parameters of the vinegar [5,6,7,28].

Rice, grapes, apples, malt, honey, and potatoes are most used in the production of vinegars [1]. However, many manufacturers of fermented foods, because of their desire to create a product combining a valuable composition with satisfactory sensory qualities, are introducing ever newer variants of vinegars, obtained by using a variety of raw materials such as pear, lemon, or rhubarb, to the consumer market.

Unfortunately, to date, few scientific articles have been published verifying the properties and effects of vinegars made from raw materials other than rice, grapes, apples, malt, honey, and potatoes [29,30,31,32]. In the literature, there is also a lack of data on the commercial live vinegars available on the Polish food market.

Therefore, the aim of this study was to analyze the chemical composition and antioxidant properties of the selected live fruit or vegetable vinegars available on the Polish food market, as well as to compare and select the best product in terms of its potential health benefits. To the best of our knowledge, this is the first publication in which rhubarb, lemon, and pear vinegars were analyzed.

## 2. Materials and Methods

### 2.1. Material

The research material consisted of four live (naturally turbid, unfiltered, unpasteurized) fruit and vegetable vinegars from a producer of organic preserves (Delikatna.bio, Bacillus SP. z o.o., Gdynia, Poland), available on the Polish food market. Live vinegars were characterized by having a typical slightly acidic, yet balanced flavor. The taste and aroma of the fruit or vegetable which the vinegar was produced from were noticeable.

### 2.2. The Determination of the Total Polyphenol Content (TPC)

The determination of the total polyphenol content (TPC) was performed according to the Singleton V.L., Rossi J.A method using the Folin—Ciocalteu reagent (Chempur, Poland) [33]. An amount of 5.0 mL of a 10% Folin—Ciocalteu aqueous solution and 1.0 mL of a test sample were added into the vial. The vial contents were thoroughly mixed and after 5 min, 4.0 mL of a 7.5% Na_2_CO_3_ solution was added and incubated for 60 min at room temperature. The reference solution was prepared the same way but instead of the tested sample, distilled water was added. Absorbance was measured at 765 nm (8453UV, AGILENT TECHNOLOGIES, Santa Clara, CA, USA). All assays were performed in nine replicates. The content of polyphenols was determined from the calibration curve using gallic acid (GAE) as the reference standard (0–200 mg/L of gallic acid). The results are shown in mg/L gallic acid [34].

### 2.3. The Determination of the Total Flavonoid Content (TFC)

The determination of the total flavonoid content was performed according to the Pękal and Pyrzynska and Hu methods [35,36]. Firstly, 0.6 mL of a 5% NaNO_2_ aqueous solution and 2.0 mL of a test sample were added into the vial. The vial contents were thoroughly mixed and incubated for 6 min, then 0.5 mL of a 10% AlCl_3_ aqueous solution was added. Incubation was repeated under the same conditions. Then, 3.0 mL of an NaOH aqueous solution (4.3%) and 3.9 mL of distilled water were added to the vial. The contents of the vial were again mixed thoroughly and incubated for 15 min. Absorbance was measured at 510 nm (8453UV, AGILENT TECHNOLOGIES, Santa Clara, CA, USA). All assays were performed in nine replicates. The content of flavonoids was determined from the calibration curve using the rutin equivalent as the reference standard (0–120 mg/L of rutin equivalent) [34].

### 2.4. The Determination of the Ferric Ion Reducing Antioxidant Power (FRAP) Method

The determination of the ferric ion reducing antioxidant power was performed according to the methods of Benzie and Strain [37,38]. A FRAP solution was prepared by mixing (10:1:1 *v*/*v*/*v*) 300 mM of an acetate buffer (pH = 3.6), 10 mM of a 2,4,6-Tris(2-pyridyl)-s-triazine (TPTZ) solution in 40 mM of HCl, and 20 mM of a FeCl_3_ aqueous solution. An amount of 3 mL of the FRAP reagent, 0.1 mL of the test sample, and 0.3 mL of distilled water were added into the vial. The vial contents were thoroughly mixed and placed at 37 °C for 5 min. The absorbance was measured at 593 nm (8453UV, AGILENT TECHNOLOGIES, Santa Clara, CA, USA). All assays were performed in nine replicates. The ferric ion reducing antioxidant power was determined from the calibration curve using Fe (II)/L as the reference standard (0–5000 µM Fe (II)/L). The results were expressed as µM Fe (II)/L [34].

### 2.5. Antioxidant Activity by the DPPH Methods

The antioxidant activity of samples was measured with the spectrophotometric method using synthetic radical DPPH (2.2-diphenyl-1-picrylhydrazyl, Sigma, Poznań, Poland) according to the methods of Brand-Williams et al. and Pekkarinen et al. [39,40]. The DPPH stock solution was prepared on the day of analysis by dissolving 0.01183 g of DPPH in 96% ethanol. The DPPH solution was diluted with ethanol to obtain an absorbance of 1.0 ± 0.02 at 518 nm. Then, 3.9 mL of a DPPH ethanolic solution, and 0.1 mL of the test sample were added into the vial. The vial contents were thoroughly mixed and incubated in the dark for 30 min at room temperature. The control solution was prepared in the same way but instead of the tested sample, 96% ethanol was added. The spectral absorbance was immediately measured at 518 nm (8453UV, AGILENT TECHNOLOGIES, Santa Clara, CA, USA). All assays were performed in nine replicates. The results are shown in the % of DPPH radical inhibition [41].

The antioxidant potential (antioxidant activity, inhibition) of the tested solutions has been expressed by the percent of DPPH inhibition, using the following formula:

$$\%\;inhibition\;=(A_{0}\;-\;A_{s})/A_{0}\;\times \;100\;$$

where:

A_0_—the absorbance of the control DPPH solution at 518 nm
A_s_—the absorbance of the tested DPPH solution at 518 nm

### 2.6. Antioxidant Activity via ABTS Methods

The antioxidant activity of samples was measured with the spectrophotometric method using an ABTS reagent (2,2′-azobis(3-ethylbenzothiazolin-6-sulfonate, SIGMA-ALDRICH, Poznań, Poland). An ABTS stock solution was prepared by mixing 5 mL of 7 mM of an ABTS aqueous solution and 5 mL of 2.45 mM of an potassium persulfate (K_2_S_2_O_8_) aqueous solution, and was incubated in the dark for 12–16 h. The ABTS stock solution was diluted with ethanol to obtain an absorbance of 1.0 ± 0.02 at 750 nm. Then, 0.1 mL of the tested sample and 2.9 mL of the ABTS reagent were added into the vial. The vial contents were thoroughly mixed and incubated in the dark for 6 min at room temperature. The spectral absorbance was immediately measured at 750 nm (8453UV, AGILENT TECHNOLOGIES, Santa Clara, CA, USA). All assays were performed in nine replicates. The results are shown in the % of ABTS radical inhibition [41].

The antioxidant potential (antioxidant activity, inhibition) of tested solutions has been expressed by the percent of ABTS inhibition, using the following formula:

$$\%\;inhibition\;=\;(A_{0}\;-\;A_{s})/A_{0}\;\times \;100\;$$

where:

A_0_—the absorbance of the control ABTS solution at 750 nm
A_s_—the absorbance of the tested ABTS solution at 750 nm

### 2.7. The Determination of the Vitamin C Content

In this method, 2,6-dichlorophenoloindophenol (2,6-DCPIP (SIGMA-ALDRICH, Poznań, Poland) is added to a sample, reacts with vitamin C, and after extraction with xylene (SIGMA-ALDRICH, Poznań, Poland), its excess is determined spectrophotometrically. Absorbance measurements were taken at 500 nm in 1 cm quartz cuvettes with xylene as a reference. Measurements were taken on an Agilent 8453 UV-VIS spectrophotometer [34]. The concentration of vitamin C was expressed in the mg of vitamin C per 100 mL of vinegar (mg/100 mL).

### 2.8. The Determination of pH

The pH of the vinegars was determined using a pH meter (SCHOTT Instruments; SI Analytics Mainz, Mainz, Germany) [34].

### 2.9. The Determination of Acetic Acid

Chromatographic analyses were conducted using the Agilent Technologies 1260 A GC system with a flame ionization detector (FID). A fused-silica capillary column with a free fatty acid phase (DB-FFAP, 30 m × 0.53 mm × 0.5 μm) was used. The carrier gas was hydrogen at a flow rate equal to 14.4 mL/min. The initial temperature (100 °C) was maintained for 0.5 min, then raised to 180 °C with a ramping of 8 °C/min to be constant for 1 min. Subsequently, the temperature was increased to 200 °C (ramping 20 °C/min) to eventually reach 200 °C and be sustained for 5 min. The injection volume was 5 μL, and the run time of a single analysis was 17.5 min [42]. Results were presented as a percentage of the acid content, according to the surface area. Moreover, the amount of acetic acid was evaluated using the calibration curve method (mM of acetic acid/L).

### 2.10. The Determination of the Flavonoids and Phenolic Acids

Liquid chromatography (Agilent Technologies 1260 HPLC System, Santa Clara, CA, USA) was used in the order to determinate the polyphenol compounds. The column used was Hypersil Gold (150 × 4.6) with the temperature maintained at 25 °C. The detection of phenolic compounds was performed via UV absorption at λ = 278 nm. Each compound was identified based on its retention time and by comparison with standards under the same conditions. The mobile phase consisted of a 1% aqueous acetic acid solution (A) and 100% MeOH (B). The samples were eluted with the following gradient: 90% A and 10% B from 0 to 6 min, 84% A and 16% B from 7 to 25 min, 72% A and 28% B from 26 to 37 min, 65% A and 35% B from 38 to 47 min, 50% A and 50% B from 48 to 64 min, and 90% A and 10% B from 65 to 70 min, to restore the initial conditions, before the injection of a new sample. The flow rate was 0.8 mL/min, and the injection volume was 30 µL.

### 2.11. Statistical Analysis

Statistical analysis was conducted utilizing MedCalc **^®^** Statistical Software version 20.218 (MedCalc Software Ltd., Ostend, Belgium; https://www.medcalc.org; accessed on 28 February 2024) and Microsoft Excel 2017. Results were presented using median, upper, and lower quartile, minimum, and maximum values. To assess the differences among the study parameters, we employed the non-parametric Kruskal–Wallis test with Conover’s post hoc test. Spearman’s test was utilized for calculating correlation coefficients. Statistical significance was considered at *p* ≤ 0.05.

## 3. Results

### 3.1. The Analysis of the Antioxidant Properties and Phytochemical Compositions of the Vinegars

The antioxidant activity of the tested fruit and vegetable vinegars was evaluated through their capacity for ferric ion reducing (FRAP) and ability to neutralize radicals (DPPH, ABTS).

**Figure 1:**
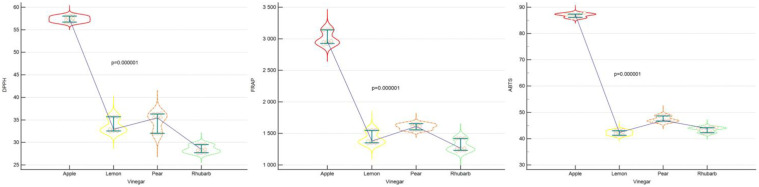
Box plots of the antioxidant potentials (expressed by median, upper, and lower quartiles, minimum and maximum values) of the vinegars tested using the DPPH, FRAP, and ABTS methods.

The antioxidant potential values of the examined vinegars, expressed as the ability to reduce iron ions, ranged from 1264.40 to 2962.30 µM Fe (II)/L. The apple vinegar had the highest recorded value, while the pear vinegar demonstrated the lowest (Figure 1). Statistically significant differences occurred between all vinegars (Table 1).

The antioxidant potential values of the vinegars tested, quantified as percentages of ABTS radical inhibition, varied between 42.35 and 87.23%. The apple vinegar had the highest recorded value, while the rhubarb vinegar had the lowest (Figure 1). Statistically significant differences were observed between all vinegars (Table 2).

The antioxidant potential values of the vinegars tested, quantified as percentages of DPPH radical inhibition, varied between 28.43 and 57.40%. The apple vinegar had the highest recorded value, while the pear vinegar had the lowest (Figure 1). Statistically significant differences were observed between all vinegars except for the rhubarb vinegar vs. the lemon vinegar (Table 3).

The phytochemical composition of the vinegars was assessed by measuring the contents of the polyphenols (TPCs), flavonoids (TFCs), and vitamin C.

The total polyphenol contents (TPCs) of the vinegars tested ranged from 61.05 to 191.97 mg GAE/L. The apple vinegar exhibited the highest values for this parameter, whereas the pear vinegar had the lowest. Statistically significant differences were observed between all vinegars except for the rhubarb vinegar vs. the lemon vinegar (Table 4).

The total flavonoid contents (TFCs) of the examined vinegars ranged from 40.58 to 70.22 mg RE/L. The apple vinegar had the highest total flavonoid content, while the rhubarb vinegar demonstrated the lowest. Statistically significant differences were observed between all vinegars except for the pear vinegar vs. the lemon vinegar (Table 5).

An analysis of the quantitative and qualitative compositions of polyphenolic compounds in the vinegars showed the presence of 11 compounds, including phenolic acids: chlorogenic acid, 4-hydroxybenzoic acid, 2-hydroxycinnamic acid, synapinic acid, ellagic acid, dihydroxybenzoic acid, and caffeic acid; and flavonoids: resveratrol, myricetin, kemferol, and apigenin. Among the phenolic acids, the tested products contained the highest amounts of 4-hydroxybenzoic acid, chlorogenic acid, and caffeic acid, while among flavonoids they contained the highest amounts of resveratrol and myricetin. The content of the identified compounds differed statistically significantly between the tested vinegars (Table 6 and Table 7). The highest content of sinapic acid (0.967 mg/L) and kaempferol (0.485 mg/L) was detected in the lemon vinegar. The occurrence of apigenin was only recorded in the lemon vinegar (0.156 mg/L). The rhubarb vinegar showed the highest content of resveratrol (12.711 mg/L), myricetin (7.447 mg/L), chlorogenic acid (21.273 mg/L), ellagic acid (0.455 mg/L), dihydroxybenzoic acid (9.238 mg/L), caffeic acid (10.473 mg/L) and, together with the apple vinegar, showed the highest content of 4-hydroxybenzoic acid.

Vinegars also appear to be a good source of vitamin C. Its highest concentration was identified in the lemon vinegar, at 15.95 mg/100 mL, while the lowest concentration was found in the pear vinegar, at 10.47 mg/100 mL, and in the apple vinegar, at 10.89 mg/100 mL. Statistically significant differences were observed between all vinegars except for the pear vinegar vs. the apple vinegar (Table 8).

**Figure 2:**
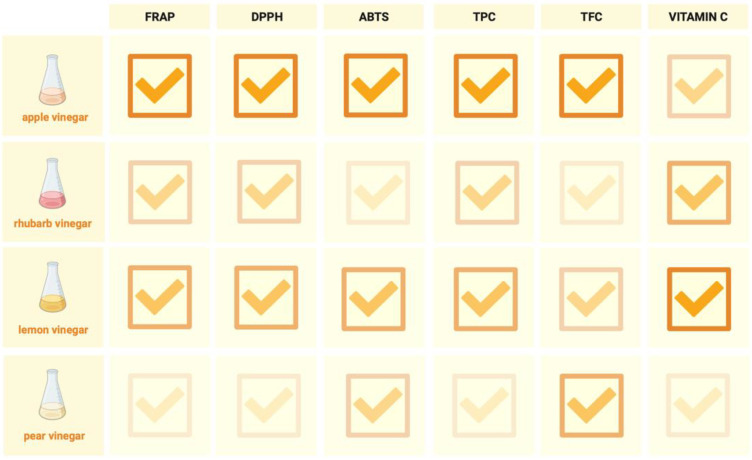
The comparison of the antioxidant properties of the tested vinegars. The values of the tested parameters were determined by changing the color intensity.

The apple vinegar performed best in terms of its antioxidant properties (Figure 2). The antioxidant content, both that of polyphenols and flavonoids, of the apple vinegar was significantly higher than that of the other vinegars: the lemon, pear, and rhubarb. It also showed the highest antioxidant potential tested by all methods: ABTS, FRAP, and DPPH. In contrast, the lemon vinegar proved to be the best source of vitamin C.

### 3.2. The Analysis of the pH and Acetic Acid Contents of the Vinegars

The pH values of the vinegars tested ranged from 3.14 to 3.41. The highest pH was found in the pear vinegar and the apple vinegar, and the lowest was that in the rhubarb vinegar. No statistically significant differences were observed for the apple vinegar vs. the lemon vinegar and the apple vinegar vs. the pear vinegar (Table 9).

The acetic acid content of the vinegars tested ranged from 29.180 to 38.125 mM/L. The rhubarb vinegar contained the most acetic acid, while the pear vinegar contained the least. There were no statistically significant differences for the apple vinegar vs. the lemon vinegar (Table 10).

**Figure 3:**
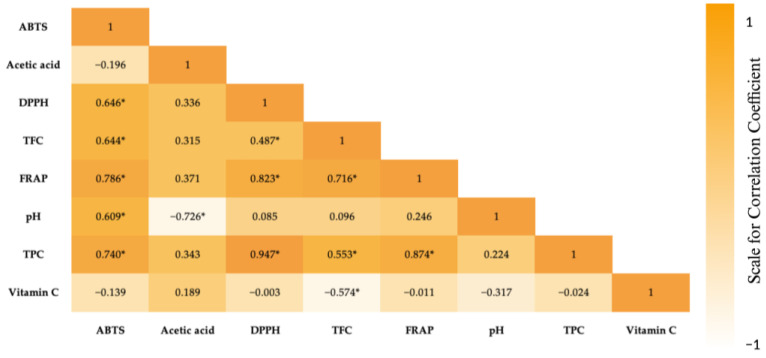
Spearman’s rank correlations between the tested parameters for the different types of vinegars. *—statistically significant (*p* ≤ 0.05).

The statistical analysis of the results showed statistically significant (for *p* ≤ 0.05) correlations between the tested parameters of the vinegars, which are presented in Figure 3. A highly significant positive correlation, with a coefficient value of r = 0.947, was identified between the concentration of polyphenols and the antioxidant potentials as assessed by the DPPH method. Furthermore, strong positive correlations, with a coefficient value of r = 0.874, were observed between the polyphenol contents and antioxidant potentials determined via ferric ion reducing ability (FRAP). A negative correlation with a coefficient value of r = −0.726 occurred between the pH values and acetic acid concentrations.

## 4. Discussion

Vinegar is a fermented product that, thanks to its distinctive sensory qualities, has been held in great esteem in the culinary world for centuries, usually as a food fixative or seasoning. Because of its sour, intense flavor, and pungent, distinctive aroma, it is commonly known as an ingredient in dressings and marinades [43]. Furthermore, in recent years, it has also become known as a functional food through the increased interest in its health-promoting properties. Scientists, recognizing the huge therapeutic potential in vinegars, are constantly looking for newer and newer applications for them.

Although, traditionally, rice, grapes, apples, malt, honey, and potatoes have been used in the production of vinegars, other substrates, which are sources of carbohydrates, are increasingly being used in their production. Promising raw materials in terms of their nutritional values are fruits and vegetables such as lemons, rhubarbs, and pears. Lemons are characterized by a rich chemical composition, dominated by polyphenols, such as flavonoids and phenolic acids, in addition to coumarin compounds, amino acids, carbohydrates, vitamins (C, A, E, B), and bioelements (potassium, calcium, magnesium, sodium) [44,45,46]. Rhubarb is a source of vitamins (A, C, E, B_9_), minerals (phosphorus, potassium), dietary fiber, and polyphenolic compounds, including rapontygenin, which exhibits anti-allergic and anti-cancer effects. On the other hand, pears contain, among other vitamins (C, A, K, B), minerals (calcium, magnesium, phosphorus, iron, copper, iodine), dietary fibers, and several compounds with antioxidant properties [47,48]. Given that the fermentation process potentiates the bioavailability of some nutrients contained in the raw materials, lemon-, rhubarb- and pear-based vinegars can be products with nutritional compositions and beneficial antioxidant properties, as first studied in this paper.

It has been shown that live vinegars, regardless of the base raw material, exhibit strong antioxidant properties and thus a high capacity to counteract the harmful effects of free radicals. This discovery is extremely optimistic for people exposed to pro-oxidant–antioxidant imbalances, which can contribute to the development of obesity, diabetes atherosclerosis, as well as malignancies, among others. The diseases of civilization are one of the biggest health problems of the 21st century. Worldwide, according to WHO data, up to 41 million people die from them annually [49]. The reported figures show the enormous scale of the problem. One way to curb the escalation of this pandemic is prevention. The results of numerous scientific studies show that long-term adherence to diets rich in antioxidants correlates with a lower incidence of free-radical chronic diseases and associated mortality rates [50,51,52]. Thus, incorporating a widely available product such as vinegar into the diet seems a viable idea for an innovative preventive regimen.

A detailed analysis of the antioxidant properties of lemon, rhubarb, pear, and apple vinegars was carried out by measuring the vinegars’ ferric ion reducing ability (FRAP) and neutralizing radicals (DPPH, ABTS).

Antioxidant potential values, expressed as a percentage of ABTS radical inhibition, ranged from 42.35 to 87.23%. The apple vinegar had the highest recorded value, while the rhubarb vinegar had the lowest. The measurement made by the DPPH method, in which the results obtained ranged from 28.43 to 57.40%, positively correlated with the FRAP method. The antioxidant potential of the sourdoughs measured by the FRAP method ranged from 1264.40 to 2962.30 µM Fe (II)/L. The apple vinegar had the highest recorded value in both methods, while the pear vinegar had the lowest. The observed difference in the obtained results may be due to the selectivity of each method. The ABTS and FRAP methods allow for the determination of the antioxidant capacity of both hydrophilic and lipophilic compounds [53]. In contrast, DPPH is soluble only in organic solvents, making it impossible to measure the antioxidant activity of hydrophilic compounds [54]. Therefore, slightly lower values were recorded in the use of this method.

Jo et al. analyzed the antioxidant potential of commercially available apple vinegars. They showed that the values of this parameter measured by the ABTS method ranged from 11.78% to 99.61%, while those determined by the DPPH method ranged from 16.18 to 57.67% [55]. There are few studies describing the antioxidant properties of commercial vinegars based on other fruits or vegetables. One of them is a study by Hamden et al. in which they examined commercial date vinegar. According to their results, a free radical scavenging capacity measured by the DPPH method of 0.62 mg TE/mL and 0.58 µmol AEAC/mL measured by the FRAP method were shown [30]. According to Lee et al., commercial pomegranate vinegar, onion vinegar, and apple vinegar showed 92.13, 33.05, and 2.91% DPPH free radical scavenging capacities and 98.43, 63.27, and 5.85% ABTS radical scavenging capacities, respectively [56]. The quoted research results show how important the type of raw material used is for the antioxidant properties of the vinegar.

The phytochemical composition of the tested vinegars was assessed by measuring the total polyphenols (TPCs), and the total flavonoids (TFCs).

All vinegars had high polyphenol contents from 61.05 to 191.97 mg GAE/L and flavonoids from 40.58 to 70.22 mg RE/L. In both cases, the apple vinegar showed the highest values. Liu et al. also conducted a study in which they analyzed the chemical profile of 23 fruit vinegars available on the Chinese food market. The vinegars studied differed significantly in the amount of phytonutrients present in them. In the apple vinegars, of which there were 11, the polyphenol content ranged from 43.75 to 495.52 mg GAE/L, while the flavonoids ranged from 2.22 to 31.39 mg QE/L [29]. Another group of researchers, aiming to identify a method conducive to the better quality of the obtained products, studied and compared, among other things, the antioxidant properties of artisanal and industrially produced apple vinegar. Both the higher contents of polyphenols (106.91 mg GAE/L) and flavonoids (11.36 mg RE/L) were characterized by the apple vinegar obtained via the artisanal method [57]. These results are similar to those obtained in our study. Some discrepancies may be due to, among other things, the use of other apple varieties or the use of a pasteurization or filtration process for the vinegars. Antoniewicz et al. conducted a study aimed at, among other things, assessing the total polyphenol content in live grape vinegars produced from five different grape varieties through spontaneous fermentation with or without the addition of sugar. The total polyphenol content in the grape vinegars was highest in the vinegar produced from Prior grapes with added sugar (1437.77 ± 14.74 mg GAE/L), and lowest in the vinegar produced from Solaris grapes without added sugar (289.8 ± 38.04 mg GAE/L). The results of this group of scientists’ study demonstrated that both the grape variety and the addition of sugar in the vinegar production process significantly influenced the polyphenol content in the final product [58]. Kahle et al. analyzed the polyphenol contents of freshly prepared juices from different varieties of dessert apples. The values obtained ranged from 154 to 178 mg/L [59]. The higher content of phytochemicals in vinegars is, among other things, the result of the metabolic activity of microorganisms [60,61,62]. Some bacteria and yeasts included in starter cultures show the ability to produce and release enzymes (e.g., invertase) that break down complex polyphenolic compounds into smaller molecules with higher biological activities [63,64]. Moreover, acids produced during acetic fermentation can degrade the glycosidic bonds of phenolic compounds, leading to the release of other bioactive compounds [56,65]. Therefore, there may be an increase in the total polyphenol content as a result of the vinegar production process. However, some of them may also polymerize, transforming into higher molecular weight molecules, thereby reducing the overall antioxidant content [66]. It is also worth noting that the polyphenol content (TPC) in the tested vinegars correlated strongly positively with the antioxidant activity as assessed by the DPPH and FRAP methods, confirming that these compounds have antioxidant potential.

Liu et al. showed that the most common phenolic compounds in fruit vinegars are gallic acid, protocatechuic acid, chlorogenic acid, caffeic acid, and p-coumaric acid [29]. Kašpar et al. came to a similar conclusion, as they analyzed 14 samples of balsamic vinegars purchased from Czech e-stores and local markets and found that the main phenolic compounds found in balsamic vinegars were gallic acid, protocatechuic acid, caffeic acid, and p-coumaric acid [27]. On the other hand, Bakir et al., as a result of an HPLC analysis of the polyphenolic profile of 18 different vinegars, showed that gallic acid, protocatechuic acid, and p -hydroxybenzoic acid were the most common compounds present [67]. In the 4 vinegars analyzed in this study, we identified 11 polyphenolic compounds, including phenolic acids: chlorogenic acid, 4-hydroxybenzoic acid, 2-hydroxycinnamic acid, sinapic acid, ellagic acid, dihydroxybenzoic acid, caffeic acid; and flavonoids: resveratrol, myricetin, kempferol and apigenin. Among the phenolic acids, the tested products contained the highest amounts of 4-hydroxybenzoic acid, chlorogenic acid, and caffeic acid, while among the flavonoids, they contained resveratrol and myricetin in the highest amounts. All vinegars tested contained dihydroxybenzoic acid, 4-hydroxybenzoic acid, caffeic acid, 2-hydroxycinnamic acid, and myricetin. Apigenin, which exhibits a range of health-promoting properties including antibacterial, antiviral, antiproliferative, anti-inflammatory, antioxidant, anti-angiogenic, and anticancer activities, was only contained in apple vinegar [68]. Sinapic acid, which is a compound with multiple health benefits, i.e., antioxidant, anti-inflammatory, anticancer, antimutagenic, antiglycaemic, neuroprotective, and antimicrobial effects, was only found in two of the vinegars: the lemon vinegar and the pear vinegar [69]. In contrast, the rhubarb vinegar showed the highest content of resveratrol (12.711 mg/L), myricetin (7.447 mg/L), chlorogenic acid (21.273 mg/L), ellagic acid (0.455 mg/L), dihydroxybenzoic acid (9.238 mg/L), caffeic acid (10.473 mg/L) and, together with the apple vinegar, showed the highest content of 4-hydroxybenzoic acid. The identified phytonutrients in the rhubarb vinegar may positively affect the functioning of the body, as they exhibit antioxidant, anti-inflammatory, anti-diabetic, anti-cancer, cardioprotective, prebiotic effects, as well as beneficial effects on cognitive function, contributing to the reduction of neurodegenerative diseases [70,71,72,73,74]. The pear vinegar was the only one that did not contain chlorogenic acid, ellagic acid, resveratrol, as well as kempferol, which may mean that it is a less valuable product in terms of the diversity of its polyphenolic compounds and, as a result, may translate into a weaker antioxidant effect for the consumer’s body.

Vinegars also present themselves as promising reservoirs of vitamin C. Ascorbic acid, possessing robust antioxidant properties, effectively scavenges free radicals, thereby conferring protection against oxidative stress upon the body. Moreover, it augments iron absorption, contributes to the synthesis of neurotransmitters, thereby enhancing cognitive function, promotes wound healing, participates in collagen synthesis, prevents and shortens infections, and exerts a beneficial impact on the cardiovascular system by mitigating the risk of ischemic heart disease and other cardiovascular disorders [75,76,77,78]. Since the human body does not have the ability to synthesize and store it, it must be supplied in adequate amounts with food. In the products studied, its content ranged from 10.47 to 15.95 mg/100 mL. Another group of researchers reported that the content of vitamin C in apple vinegar, depending on the production method, is 13.64–15.4 mg/100mL [57]. Considering that the recommended daily allowance (RDA) for vitamin C is 75 mg for women and 90 mg for men, consuming 100 mL of lemon vinegar can cover 21.27% of the requirement for women and 17.72% of that for men [79].

Vinegars are also a source of acetic acid (AA). The content of AA varies depending on the type of vinegar. Nonetheless, in accordance with the guidelines established by the United States Food and Drug Administration (FDA), to ensure product safety and quality, the quantity of acetic acid should not be lower than 4%. The vinegars tested in this study contained from 29.180 to 38.125 mM of acetic acid. This compound gives them specific sensory characteristics so that they can be identified by the senses. It is an effective antiseptic and is responsible for many of the therapeutic properties of vinegar [80]. It has been suggested that, through several mechanisms, it can lower fasting glucose levels, promote weight loss, and regulate blood pressure [81,82,83]. Furthermore, their acetic acid content determines the low pH of the vinegars, so there was a strong negative correlation between these parameters. The pH values of the vinegars analyzed ranged from 3.14 to 3.41, which was consistent with the results of other authors [67,84]. The lowest pH (3.14) and, at the same time, the highest acetic acid content (38.125 mM/L) was identified in the rhubarb vinegar. It is worth noting that a low pH offers microbiological safety, as only a limited number of pathogenic microorganisms can thrive in this environment. Furthermore, the acidic conditions generated during the fermentation process contribute positively to the stability of vitamin C [85,86]. However, from a medical perspective, too much vinegar in the diet can adversely affect the gastrointestinal tract and lead to an acid–base imbalance [87,88]. Therefore, to avoid undesirable effects, it is advisable to consume vinegar in the amounts recommended by the manufacturer (1–2 tbsp/day).

It is also worth emphasizing that the fermentation process is not only a beneficial way of processing vegetables or fruits in terms of increasing their nutritional value, but also a way of preventing the waste of seasonal foods with a short shelf life. It is estimated that fruits and vegetables are among the most consumed commodities in the world and account for more than 42% of total food waste [89]. Statistically, about 3.7 billion apples end up in landfills each year [90]. Although some alternatives to direct consumption, i.e., the production of jams or fruit concentrates, have already been introduced, a large amount is still thrown away, generating large economic losses, as well as the accumulation of organic waste. This means that the production of vinegars from fruits as well as vegetables carries multidimensional profits.

## 5. Conclusions

Live vinegars have high antioxidant potentials and are a good source of antioxidants in the diet. All of them contained dihydroxybenzoic acid, 4-hydroxybenzoic acid, caffeic acid, 2-hydroxycinnamic acid, and myricetin. The lemon vinegar showed the highest vitamin C content and the greatest variety of polyphenolic compounds. The apple vinegar appeared to be the best source of polyphenols and flavonoids and therefore the most promising nutraceutical. Thus, it appears to be the best nutritional choice with potentially beneficial health-promoting properties.

## Figures and Tables

**Table 1:** The antioxidant potentials of the vinegars tested using the FRAP method. Data represent the minimum, 25th percentile, median, 75th percentile, and maximum values of three replicates. Statistically significant differences (*p* ≤ 0.05).

FRAP (µM Fe (II)/L)						
Vinegar Type	Minimum	25th Percentile	Median	75th Percentile	Maximum	K–W *p* Value
Apple vinegar ^a^	2907.80	2928.30	2962.30 *^b,c,d^	3140.53	3152.10	<0.001
Rhubarb vinegar ^b^	1342.10	1349.90	1379.50 *^a,c,d^	1550.60	1556.00	
Lemon vinegar ^c^	1549.70	1555.75	1611.70 *^a,b,d^	1655.35	1660.50	
Pear vinegar ^d^	1224.40	1232.08	1264.40 *^a,b,c^	1419.73	1427.20	

K–W—Kruskall–Wallis; Different letters (a–d) represent different types of vinegars: a—Apple vinegar, b—Rhubarb vinegar, c—Lemon vinegar, d—Pear vinegar. Letters in the superscript mean that the results differ significantly at a * *p* < 0.05 in the post hoc test.

**Table 2:** The antioxidant potentials of the vinegars tested using the ABTS method. Data represent the minimum, 25th percentile, median, 75th percentile, and maximum values of three replicates. Statistically significant differences (*p* ≤ 0.05).

ABTS (%)						
Vinegar Type	Minimum	25th Percentile	Median	75th Percentile	Maximum	K–W *p* Value
Apple vinegar ^a^	85.76	86.12	87.23 *^b,c,d^	87.42	87.87	<0.001
Rhubarb vinegar ^b^	41.12	41.32	42.35 *^a,c,d^	43.05	43.34	
Lemon vinegar ^c^	46.33	46.71	46.92 *^a,b,d^	48.64	48.90	
Pear vinegar ^d^	42.14	42.31	43.89 *^a,b,c^	44.15	44.26	

K–W—Kruskall–Wallis; Different letters (a–d) represent different types of vinegars: a—Apple vinegar, b—Rhubarb vinegar, c—Lemon vinegar, d—Pear vinegar. Letters in the superscript mean that the results differ significantly at a * *p* < 0.05 in the post hoc test.

**Table 3:** The antioxidant potentials of the vinegars tested using the DPPH method. Data represent the minimum, 25th percentile, median, 75th percentile, and maximum values of three replicates. Statistically significant differences (*p* ≤ 0.05).

DPPH (%)						
Vinegar Type	Minimum	25th Percentile	Median	75th Percentile	Maximum	K–W *p* Value
Apple vinegar ^a^	56.62	56.71	57.40 *^b,c,d^	58.04	58.22	<0.001
Rhubarb vinegar ^b^	32.44	32.56	32.94 *^a,d^	35.68	35.71	
Lemon vinegar ^c^	31.97	32.04	35.48 *^a,d^	36.33	36.47	
Pear vinegar ^d^	27.64	27.71	28.43 *^a,b,c^	29.56	29.61	

K–W—Kruskall–Wallis; Different letters (a–d) represent different types of vinegars: a—Apple vinegar, b—Rhubarb vinegar, c—Lemon vinegar, d—Pear vinegar. Letters in the superscript mean that the results differ significantly at a * *p* < 0.05 in the post hoc test.

**Table 4:** The total polyphenol contents (TPCs) in the vinegars. Data represent the minimum, 25th percentile, median, 75th percentile, and maximum values of three replicates. Statistically significant differences (*p* ≤ 0.05).

TPC (mg GAE/L)						
Vinegar Type	Minimum	25th Percentile	Median	75th Percentile	Maximum	K–W *p* Value
Apple vinegar ^a^	188.82	189.10	191.97 *^b,c,d^	192.06	192.08	<0.001
Rhubarb vinegar ^b^	92.46	92.49	93.81 *^a,d^	95.05	95.19	
Lemon vinegar ^c^	93.09	93.13	94.63 *^a,d^	95.24	95.26	
Pear vinegar ^d^	59.94	60.00	61.05 *^a,b,c^	61.72	61.74	

K–W—Kruskall–Wallis; Different letters (a–d) represent different types of vinegars: a—Apple vinegar, b—Rhubarb vinegar, c—Lemon vinegar, d—Pear vinegar. Letters in the superscript mean that the results differ significantly at a * *p* < 0.05 in the post hoc test.

**Table 5:** The total flavonoid contents (TFCs) in the vinegars. Data represent the minimum, 25th percentile, median, 75th percentile, and maximum values of three replicates. Statistically significant differences (*p* ≤ 0.05).

TFC (mg RE/L)						
Vinegar Type	Minimum	25th Percentile	Median	75th Percentile	Maximum	K–W *p* Value
Apple vinegar ^a^	59.28	59.63	70.22 *^b,c,d^	84.59	86.25	<0.001
Rhubarb vinegar ^b^	39.77	40.20	40.58 *^a,c,d^	41.27	41.61	
Lemon vinegar ^c^	41.40	41.63	41.96 *^a,b^	42.31	42.58	
Pear vinegar ^d^	37.90	39.16	43.19 *^a,b^	51.92	52.48	

K–W—Kruskall–Wallis; Different letters (a–d) represent different types of vinegars: a—Apple vinegar, b—Rhubarb vinegar, c—Lemon vinegar, d—Pear vinegar. Letters in the superscript mean that the results differ significantly at a * *p* < 0.05 in the post hoc test.

**Table 6:** The contents of phenolic acids in the vinegars. Data represent the minimum, 25th percentile, median, 75th percentile, and maximum values of three replicates. Statistically significant differences (*p* ≤ 0.05).

Compound	Vinegar Type	Minimum [mg/L]	25th Percentile [mg/L]	Median [mg/L]	75th Percentile [mg/L]	Maximum [mg/L]	K–W *p* Value
2-hydroxycinnamic acid	Apple vinegar ^a^	0.299	0.299	0.299 *^b–d^	0.306	0.308	0.0156
	Rhubarb vinegar ^b^	0.366	0.366	0.367 *^a,c,d^	0.390	0.397	
	Lemon vinegar ^c^	0.160	0.161	0.164 *^a,b,d^	0.166	0.167	
	Pear vinegar ^d^	0.260	0.262	0.265 *^a–c^	0.266	0.266	
4-hydroxybenzoic acid	Apple vinegar ^a^	19.561	19.571	19.600 *^c,d^	20.041	20.188	0.0249
	Rhubarb vinegar ^b^	21.619	21.629	21.662 *^c,d^	21.694	21.705	
	Lemon vinegar ^c^	6.401	6.433	6.531 *^a,b^	6.631	6.664	
	Pear vinegar ^d^	6.182	6.287	6.601 *^a,b^	6.611	6.614	
Sinapic acid	Apple vinegar ^a^	nd	nd	nd *^c,d^	nd	nd	0.0138
	Rhubarb vinegar ^b^	nd	nd	nd *^c,d^	nd	nd	
	Lemon vinegar ^c^	0.948	0.952	0.967 *^a,b,d^	0.982	0.987	
	Pear vinegar ^d^	0.501	0.506	0.520 *^a–c^	0.521	0.522	
Caffeic acid	Apple vinegar ^a^	9.157	9.162	9.176 *^b–d^	9.382	9.451	0.0156
	Rhubarb vinegar ^b^	10.453	10.458	10.473 *^a,c,d^	10.489	10.494	
	Lemon vinegar ^c^	1.391	1.398	1.419 *^a,b,d^	1.441	1.448	
	Pear vinegar ^d^	4.119	4.138	4.195 *^a–c^	4.201	4.203	
Dihydroxybenzoic acid	Apple vinegar ^a^	7.924	7.927	7.939 *^b–d^	8.118	8.178	0.0156
	Rhubarb vinegar ^b^	9.219	9.224	9.238 *^a,c,d^	9.252	9.256	
	Lemon vinegar ^c^	0.517	0.520	0.528 *^a,b,d^	0.536	0.538	
	Pear vinegar ^d^	1.605	1.612	1.634 *^a–c^	1.637	1.638	
Ellagic acid	Apple vinegar ^a^	0.387	0.388	0.388 *^b,d^	0.397	0.400	0.0203
	Rhubarb vinegar ^b^	0.455	0.455	0.455 *^a,c,d^	0.456	0.456	
	Lemon vinegar ^c^	0.389	0.391	0.397 *^b,d^	0.403	0.405	
	Pear vinegar ^d^	nd	nd	nd *^a–c^	nd	nd	
Chlorogenic acid	Apple vinegar ^a^	19.311	19.330	19.389 *^b–d^	19.418	19.428	0.0145
	Rhubarb vinegar ^b^	21.158	21.187	21.273 *^a,c,d^	21.305	21.315	
	Lemon vinegar ^c^	3.011	3.027	3.073 *^a,b,d^	3.120	3.136	
	Pear vinegar ^d^	nd	nd	nd *^a–c^	nd	nd	

K–W—Kruskall–Wallis; Different letters (a–d) represent different types of vinegars: a—Apple vinegar, b—Rhubarb vinegar, c—Lemon vinegar, d—Pear vinegar. Letters in the superscript mean that the results differ significantly at a * *p* < 0.05 in the post hoc test; nd—not detected.

**Table 7:** The contents of flavonoids in the vinegars. Data represent the minimum, 25th percentile, median, 75th percentile, and maximum values of three replicates. Statistically significant differences (*p* ≤ 0.05).

Compound	Vinegar Type	Minimum [mg/L]	25th Percentile [mg/L]	Median [mg/L]	75th Percentile [mg/L]	Maximum [mg/L]	K–W *p* Value
Apigenin	Apple vinegar ^a^	nd	nd	nd *^c^	nd	nd	0.0132
	Rhubarb vinegar ^b^	nd	nd	nd *^c^	nd	nd	
	Lemon vinegar ^c^	0.153	0.154	0.156 *^a,b,d^	0.158	0.159	
	Pear vinegar ^d^	nd	nd	nd *^c^	nd	nd	
Kaempferol	Apple vinegar ^a^	4.001	4.003	4.009 *^b–d^	4.099	4.129	0.0145
	Rhubarb vinegar ^b^	2.264	2.265	2.269 *^a,c,d^	2.272	2.273	
	Lemon vinegar ^c^	0.476	0.478	0.485 *^a,b,d^	0.493	0.495	
	Pear vinegar ^d^	nd	nd	nd *^a–c^	nd	nd	
Myricetin	Apple vinegar ^a^	6.035	6.057	6.123 *^b–d^	6.193	6.216	0.0156
	Rhubarb vinegar ^b^	7.432	7.436	7.447 *^a,c,d^	7.458	7.462	
	Lemon vinegar ^c^	0.740	0.744	0.755 *^a,b,d^	0.767	0.771	
	Pear vinegar ^d^	3.767	3.784	3.836 *^a–c^	3.842	3.844	
Resveratrol	Apple vinegar ^a^	11.262	11.268	11.285 *^b–d^	11.539	11.624	0.0145
	Rhubarb vinegar ^b^	12.686	12.692	12.711 *^a,c,d^	12.730	12.737	
	Lemon vinegar ^c^	1.074	1.079	1.096 *^a,b,d^	1.112	1.118	
	Pear vinegar ^d^	nd	nd	nd *^a–c^	nd	nd	

K–W—Kruskall–Wallis; Different letters (a–d) represent different types of vinegars: a—Apple vinegar, b—Rhubarb vinegar, c—Lemon vinegar, d—Pear vinegar. Letters in the superscript mean that the results differ significantly at a * *p* < 0.05 in the post hoc test; nd—not detected.

**Table 8:** The total vitamin C contents in the vinegars. Data represent the minimum, 25th percentile, median, 75th percentile, and maximum values of three replicates. Statistically significant differences (*p* ≤ 0.05).

Vitamin C (mg/100 mL)						
Vinegar Type	Minimum	25th Percentile	Median	75th Percentile	Maximum	K–W *p* Value
Apple vinegar ^a^	9.19	9.23	10.89 *^b,c^	10.98	11.08	<0.001
Rhubarb vinegar ^b^	13.94	14.10	14.66 *^a,c,d^	14.75	14.81	
Lemon vinegar ^c^	14.72	14.77	15.95 *^a,b,d^	16.48	16.52	
Pear vinegar ^d^	9.34	9.39	10.47 *^b,c^	13.91	14.02	

K–W—Kruskall–Wallis; Different letters (a–d) represent different types of vinegars: a—Apple vinegar, b—Rhubarb vinegar, c—Lemon vinegar, d—Pear vinegar. Letters in the superscript mean that the results differ significantly at * *p* < 0.05 in the post hoc test.

**Table 9:** The pHs of the vinegars. Data represent the minimum, 25th percentile, median, 75th percentile, and maximum values of three replicates. Statistically significant differences (*p* ≤ 0.05).

pH						
Vinegar Type	Minimum	25th Percentile	Median	75th Percentile	Maximum	K–W *p* Value
Apple vinegar ^a^	3.34	3.35	3.35 *^b^	3.39	3.42	0.0379
Rhubarb vinegar ^b^	3.12	3.13	3.14 *^a,c,d^	3.14	3.14	
Lemon vinegar ^c^	3.34	3.34	3.34 *^b,d^	3.35	3.35	
Pear vinegar ^d^	3.41	3.41	3.41 *^b,c^	3.41	3.41	

K–W—Kruskall–Wallis; Different letters (a–d) represent different types of vinegars: a—Apple vinegar, b—Rhubarb vinegar, c—Lemon vinegar, d—Pear vinegar. Letters in the superscript mean that the results differ significantly at a * *p* < 0.05 in the post hoc test.

**Table 10:** The acetic acid content in the vinegars. Data represent the minimum, 25th percentile, median, 75th percentile and maximum values of three replicates. Statistically significant differences (*p* ≤ 0.05).

Acetic Acid (mM/L)						
Vinegar Type	Minimum	25th Percentile	Median	75th Percentile	Maximum	K–W *p* Value
Apple vinegar ^a^	35.937	35.959	36.026 *^b,d^	36.126	36.159	0.0240
Rhubarb vinegar ^b^	37.909	37.963	38.125 *^a,c,d^	38.180	38.198	
Lemon vinegar ^c^	35.758	35.812	35.974 *^b,d^	36.054	36.080	
Pear vinegar ^d^	29.018	29.059	29.180 *^a,b,c^	29.799	30.005	

K–W—Kruskall–Wallis; Different letters (a–d) represent different types of vinegars: a—Apple vinegar, b—Rhubarb vinegar, c—Lemon vinegar, d—Pear vinegar. Letters in the superscript mean that the results differ significantly at * *p* < 0.05 in the post hoc test.

## Data Availability

The original contributions presented in the study are included in the article, further inquiries can be directed to the corresponding author.
